# The relationship between AI anxiety and work engagement among early childhood teachers: a moderated mediation model of job crafting and perceived organizational support

**DOI:** 10.3389/fpsyg.2026.1938893

**Published:** 2026-09-09

**Authors:** Haiyan Cui, Yantao Shi, Xueli Hui, Cong Zhang, Xueqian Liu

**Affiliations:** 1 Faculty of Education, Guangxi Normal University, Guilin, China; 2 College of Innovation and Entrepreneurship, Guangxi Minzu University, Nanning, China; 3 College of Preschool Education, Chongqing Youth Vocational & Technical College, Chongqing, China; 4 Foshan City Sanshui District Central Kindergarten, Foshan, China; 5 College of Education, Yuzhang Normal University, Nanchang, China

**Keywords:** AI anxiety, early childhood teachers, job crafting, perceived organizational support, work engagement

## Abstract

As artificial intelligence (AI) technology has been deeply integrated into early childhood education, the technological transformation has brought both opportunities and adaptive pressure to early childhood teachers. AI anxiety has accordingly become a prominent factor impacting teachers’ work engagement. Based on the conservation of resources theory, this study constructed a moderated mediation model to examine the association between AI anxiety and work engagement among early childhood teachers, as well as the roles of job crafting and perceived organizational support. Questionnaires were distributed to 933 early childhood teachers, and the collected data were analyzed to verify the proposed model. The results indicated that AI anxiety was negatively associated with work engagement among early childhood teachers. Job crafting mediated the link between AI anxiety and work engagement, while perceived organizational support moderated the relationship between AI anxiety and job crafting. Promoting early childhood teachers’ work engagement requires the mitigation of AI anxiety, the strengthening of perceived organizational support, and the cultivation of job crafting. This study provides empirical evidence and practical implications for alleviating AI anxiety and enhancing work engagement among early childhood teachers.

## Introduction

The rapid advancement of artificial intelligence (AI) is driving profound transformations within the education system (Chen et al., 2020). As the cornerstone of the national education system, early childhood education is experiencing the full-scale integration of AI technology. Intelligent childcare and education systems, AI interactive teaching aids, intelligent assessment tools, digital teaching support platforms, and other tools have successively entered early childhood education settings (Jin, 2019; Lee et al., 2025; Yi et al., 2024). These technologies are related to children’s development and education in terms of teaching, operations, family support, and content (Kanders et al., 2024). AI has become an important pillar for the high-quality and sustainable development of early childhood education. As educators responsible for the practical application of AI technologies, early childhood teachers are key participants in the development of the education ecosystem. Their mental state and work engagement affect the effectiveness of the transformation of early childhood education, and are crucial to the achievement of high-quality educational development.

While AI reshapes traditional educational patterns and reconstructs the education ecosystem, it has also brought teachers a host of challenges, including mental anxiety, rising workload and mounting job pressure (Hu et al., 2025). Many teachers acknowledge that the application of AI in education has great potential to save time, assist in designing diverse activities and deliver personalized learning experiences (Alwaqdani, 2025). Nevertheless, they also express concerns about the knowledge and competencies required for applying AI technologies in education, the potential risk of professional job displacement, as well as possible unintended consequences (Al-Zahrani, 2024; Aguilar-Cruz and Salas-Pilco, 2025; Moylan et al., 2025). The unease, worry or fear experienced by individuals when using AI technologies at present or in the future is defined as “AI anxiety” (Johnson and Verdicchio, 2017; Sanusi et al., 2024), which stems from computer anxiety and technophobia (Wang and Wang, 2022). AI technologies are evolving and iterating at a rapid pace, which leaves early childhood teachers anxious about the complexity of learning new technologies and the challenges to their professional identity. Different from teachers at other educational stages, early childhood teachers have unique occupational traits that render their AI anxiety more salient. The early childhood teachers in this study refer to full-time teachers who provide comprehensive childcare and educational services for children aged 3 to 6 in public and private kindergartens in China. Most of these teachers hold a humanities-oriented educational background and possess a relatively weak foundation in information technology. Amid the continuous upgrading of AI childcare and teaching tools, they face enormous pressure to update their technical capabilities, which makes them prone to intense AI anxiety. Furthermore, their daily work consists of heavy, fragmented childcare and teaching assignments alongside high-intensity emotional labor throughout the workday. Such a demanding and irregular work schedule leaves them barely any spare time for systematic AI skill learning, further aggravating their adaptation pressure amid intelligent educational transformation. As diverse AI tools are increasingly integrated into kindergarten daily work, the unfamiliar technical operations and innovative teaching application models tend to trigger negative emotions such as nervousness, worry, and psychological resistance among early childhood teachers, thereby exacerbating their AI anxiety. Existing studies have focused on the unease amid intelligent transformation and found that AI anxiety has become a prevalent psychological dilemma among early childhood teachers (Fan and Xia, 2025).

AI anxiety exerts an impact on teachers’ daily job performance, including work passion and innovative behavior (Chen et al., 2025; Zhang et al., 2025). As a key indicator for assessing teachers’ work status, AI anxiety may also be associated with work engagement. Work engagement refers to a positive, fulfilling, and work-related mindset, which is characterized by vigor, dedication and absorption (Schaufeli and Bakker, 2004; Bakker et al., 2008). Work engagement among early childhood teachers is of great significance, as it is closely associated with teachers’ job satisfaction, performance and children’s development (Mișu et al., 2022; Li et al., 2023b; Zang and Feng, 2023). Prior research has widely explored factors affecting teachers’ work engagement, which can be divided into two major aspects: personal traits and social environmental resources. Personal traits cover teacher status, gender (Topchyan and Woehler, 2021), psychological capital (Ma, 2023), teachers’ self-efficacy beliefs (Granziera and Perera, 2019), emotion regulation strategies (Zeng et al., 2025) and emotional labor (Huang and Zhou, 2024). Social environmental resources mainly include organizational climate (Zhou et al., 2026), school support (Song et al., 2013), organizational support (Yan et al., 2025), and job resources (Schaufeli and Bakker, 2004). Yet limited attention has been paid to the impact of AI anxiety triggered by AI technologies on work engagement against the backdrop of intelligent education. With the ongoing intelligent transformation of early childhood education, AI anxiety resulting from extra job demands and mental stress has become an emerging variable influencing early childhood teachers’ working status. Meanwhile, most existing studies on AI anxiety focus on corporate employees, while few studies target early childhood teachers. Exploring the correlation between AI anxiety and work engagement can not only respond to practical educational concerns in early childhood education but also enrich research concerning early childhood teachers’ occupational psychology.

According to the conservation of resources (COR) theory, individuals are inclined to conserve resources, avoid resource loss, and pursue resource gains (Hobfoll, 1989). As a positive proactive behavior through which individuals adjust work tasks, interpersonal interaction patterns and work cognition, job crafting enables people to actively reconstruct their work resources (Wrzesniewski and Dutton, 2001). Faced with the dilemma of resource depletion, teachers can adjust their working approaches and rebuild work resources via job crafting, so as to counteract the negative impacts brought by AI anxiety. However, existing research has yet to examine the mediating effect of job crafting in the relationship between AI anxiety and work engagement. Besides, COR theory emphasizes that social support resources serve as key resources for individuals to resist resource depletion and achieve resource recovery (Halbesleben et al., 2014). As an important social support resource, perceived organizational support can effectively replenish individual resources and buffer stress risks, and may play a moderating role in the relationship between AI anxiety and job crafting. Nevertheless, research concerning the roles of job crafting and perceived organizational support in the association between AI anxiety and work engagement remains to be further explored. Accordingly, based on the COR theory, this study examines the relationship between AI anxiety and work engagement of early childhood teachers, and explores the mediating role of job crafting as well as the moderating role of perceived organizational support. The present study aims to provide empirical evidence and practical strategies for alleviating teachers’ AI anxiety, optimizing organizational support systems, boosting teachers’ work engagement, and promoting the sustainable development of early childhood education.

### AI anxiety and work engagement

AI anxiety refers to an emotional response characterized by tension, worry, and unease experienced by individuals when confronting AI technology, which can be specifically categorized into anxiety about job displacement, learning anxiety, and others (Li and Huang, 2020). As AI brings transformative changes to conventional teaching, teachers need to adapt to new pedagogical demands. Since technological iteration continually outruns teachers’ professional development cycles, AI anxiety grows more evident. AI technologies can instantly generate teaching plans, conduct personalized assessments and run interactive simulations. In challenging the traditional professional authority of early childhood teachers, they also trigger teachers’ anxiety about potential job displacement and the challenges of learning new technologies. Survey respondents commented: “Parents now prefer AI-generated data reports over my professional advice” (Fan and Xia, 2025). Especially against the cultural backdrop that home-kindergarten co-education is emphasized in China, this phenomenon may further exacerbate teachers’ AI anxiety. In addition, the pace of technological advancement far exceeds teachers’ adaptive capacity, imposing substantial learning pressure on early childhood teachers during the application of AI technologies. Existing studies have shown that teachers’ anxiety negatively predicts work engagement (Liu et al., 2024). According to COR theory (Halbesleben et al., 2014), when continuous resource depletion takes place, individuals will develop negative emotions and display passive work behaviors at the initial stage. Faced with AI anxiety, early childhood teachers suffer from work-related tension. Their professional experience, occupational autonomy and psychological safety gradually decline, accompanied by the loss of psychological resources including emotions, energy and confidence. This tends to trigger resource depletion, which further diminishes teachers’ work vigor, dedication, and absorption in childcare and education, and ultimately reduces their work engagement. Accordingly, this study proposes Hypothesis 1: AI anxiety negatively predicts work engagement among early childhood teachers.

### Job crafting as a mediator

Job crafting refers to the behavior that employees proactively adjust their work tasks, cognitive styles, and interpersonal relationships to achieve a match between themselves and their work, encompassing three dimensions: task crafting, relational crafting, and cognitive crafting (Wrzesniewski and Dutton, 2001). Task crafting involves modifying the quantity, scope, type of tasks, and the allocation of personal time and energy; relational crafting entails changing the form, timing, and nature of interactions with others; and cognitive crafting refers to altering one’s perceptions of tasks and relationships. Job crafting is regarded as a positive strategy for teachers to cope with work stress (Ciuhan et al., 2022), enhance job satisfaction (Li et al., 2023a), well-being (Zhai et al., 2025), and improve work performance (Shang, 2022). It also serves as an important means for teachers to adapt to environmental changes and regain a sense of control over their work. On the one hand, AI anxiety is correlated with job crafting (Lu and Sirou, 2025). Anxiety narrows individuals’ cognitive horizons, making them more likely to perceive technology as a threat rather than a tool. Consequently, this leads to a lack of motivation to proactively integrate AI and innovate teaching methods, as well as reluctance to discuss technical difficulties with colleagues, thereby inhibiting job crafting behaviors. On the other hand, job crafting is related to work engagement (Da Silva Júnior et al., 2019). Based on COR theory (Halbesleben et al., 2014), individuals faced with resource depletion are not completely passive. They will take further proactive actions to prevent resource loss and restore resource reserves. Through job crafting, teachers can optimize the balance between job demands and resources, foster work meaning (Shang, 2022), and in turn revitalize their work engagement (Oubibi et al., 2022; Huang X. et al., 2025). Accordingly, this study proposes Hypothesis 2: Job crafting mediates the relationship between AI anxiety and work engagement.

### Perceived organizational support as a moderator

Perceived organizational support refers to employees’ overall perception that the organization values their contributions and cares about their well-being, as well as their subjective cognition and experience of whether the organization supports them (Eisenberger et al., 1986). Perceived organizational support is closely associated with employees’ positive outcomes, such as career success (Xu Z. et al., 2025) and organization-favorable outcomes (Eisenberger et al., 2020). Existing research has demonstrated that perceived organizational support is correlated with AI anxiety (Elfar, 2025). Furthermore, it is also one of the key variables associated with job crafting (Oubibi et al., 2022). These findings provide an empirical foundation for further exploring the moderating role of perceived organizational support in the relationship between AI anxiety and job crafting. From the perspective of COR theory (Halbesleben et al., 2014), perceived organizational support acts as a psychological resource that replenishes individuals’ depleted resources and mitigates the adverse impacts of negative emotions. With high organizational support, teachers can obtain professional AI training, technical support and psychological guidance, which buffers the psychological pressure of AI anxiety and weakens its negative effect on job crafting. In contrast, inadequate organizational support forces teachers to face AI challenges independently, thereby intensifying the adverse impacts of anxiety. Accordingly, this study proposes Hypothesis 3: Perceived organizational support moderates the relationship between AI anxiety and job crafting.

### The current study

**Figure 1:**
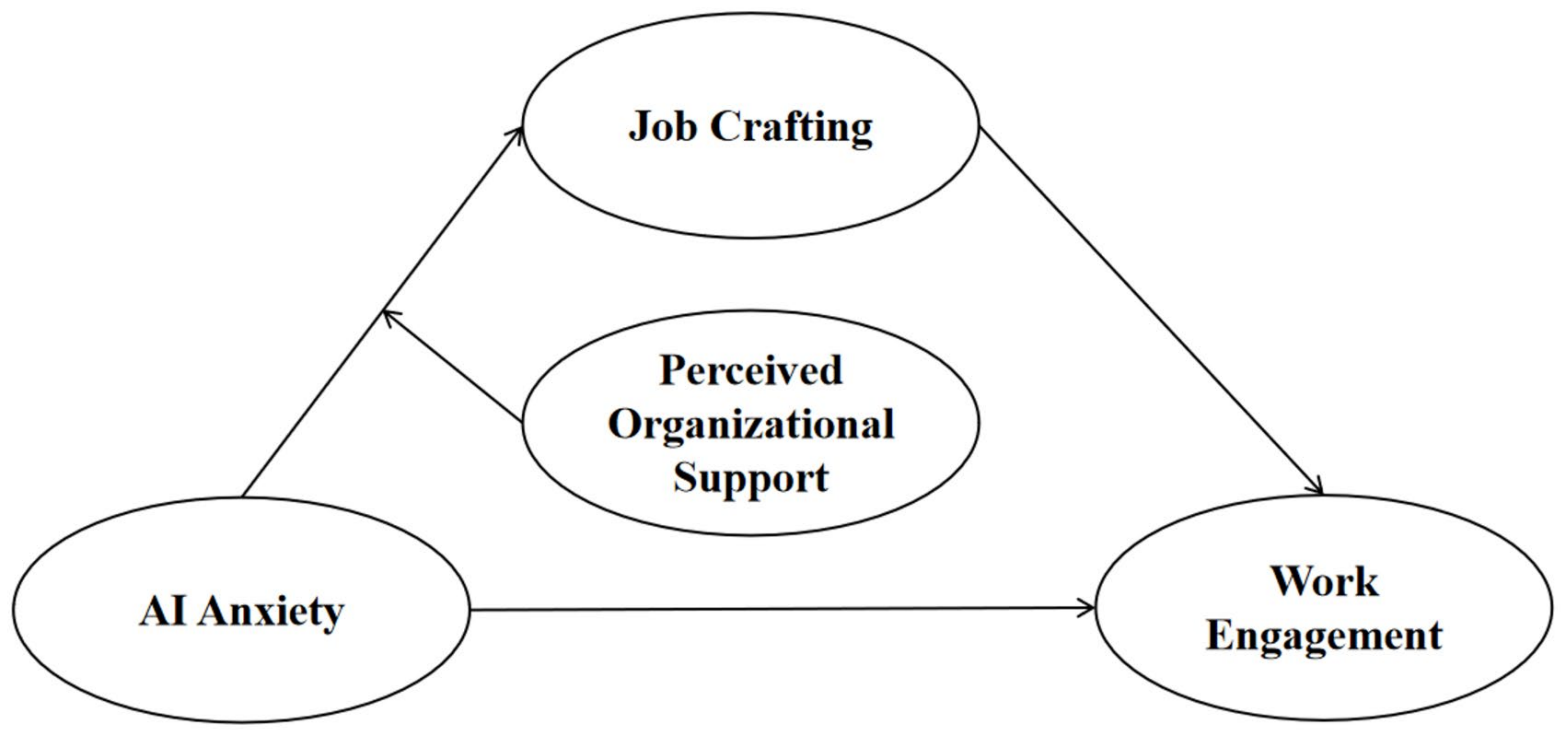
Research model.

Given the increasing stress faced by early childhood teachers and the growing attention to their well-being (Cumming, 2017), it is crucial to identify effective strategies to address the practical needs of early childhood education teacher development in the AI era, alleviate teachers’ AI anxiety, and enhance their work engagement. The mechanism underlying the impact of early childhood teachers’ AI anxiety on work engagement may be multifaceted and complex. Therefore, based on COR theory, this study intends to examine the relationship between AI anxiety and work engagement among early childhood teachers, as well as the mediating role of job crafting and the moderating role of perceived organizational support. It is expected to provide targeted theoretical and practical implications for promoting the professional development of early childhood teachers. Accordingly, this study constructs a moderated mediation model (Figure 1) and proposes the following hypotheses:

*H1*: AI anxiety negatively predicts work engagement among early childhood teachers.

*H2*: Job crafting mediates the relationship between AI anxiety and work engagement.

*H3*: Perceived organizational support moderates the relationship between AI anxiety and job crafting.

## Methods

### Participants and procedure

This study adopted a convenience sampling and recruited early childhood teachers from kindergartens in Guangzhou, Foshan, and Jiangmen, Guangdong Province, China, as research participants. The questionnaire survey was conducted through a domestic online platform. Data collection was conducted with the participants’ informed consent, and all responses were anonymous, adhering to the ethical principle of voluntary participation. Before distributing the questionnaires, the research purpose, significance, and confidentiality principles were clearly explained to the teachers to ensure that they completed the questionnaires truthfully and objectively. Samples were excluded if the questionnaire completion duration was shorter than 100 seconds or if respondents exhibited uniform response patterns (e.g., identical scores across all items). After eliminating invalid responses, 933 valid samples were retained from the initial 986 collected questionnaires, yielding an effective response rate of 94.625%.

The sample consisted of 855 females (91.640%) and 78 males (8.360%), a distribution consistent with the gender composition of early childhood teachers in reality. An analysis of the sample’s teaching experience distribution showed that 236 teachers (25.295%) had 3 years or less of teaching experience, 384 (41.158%) had 4–6 years, 204 (21.865%) had 7–9 years, and 109 (11.683%) had more than 10 years. Regarding the educational background distribution, 360 teachers (38.585%) had a college degree or below, 373 (39.979%) had a bachelor’s degree, and 200 (21.436%) had a master’s degree or above. For the type of kindergarten where the sample worked, 533 teachers (57.128%) were from public kindergartens and 400 (42.872%) from private kindergartens. As for the professional title distribution, 255 teachers (27.331%) had no professional title, 328 (35.155%) had a primary title, 288 (30.868%) had an intermediate title, and 62 (6.645%) had a senior title or above. The demographic characteristics are displayed in Table 1.

**Table 1:** Demographic characteristics of participants (*N* = 933).

Statistical variables	Group	Frequency number	Valid percentage (%)
Gender	Female	855	91.640
	Male	78	8.360
Teaching experience	3 years or less	236	25.295
	4–6 years	384	41.158
	7–9 years	204	21.865
	10 years or more	109	11.683
Educational background	College degree or below	360	38.585
	Bachelor’s degree	373	39.979
	Master’s degree or above	200	21.436
Type of kindergarten	Public	533	57.128
	Private	400	42.872
Professional title	No professional title	255	27.331
	Primary	328	35.155
	Intermediate	288	30.868
	Senior or above	62	6.645

### Measures

#### AI anxiety scale

The AI Anxiety Scale, developed and validated for the Chinese context by Li and Huang, was adopted in this study (Li and Huang, 2020). Considering the work context of early childhood teachers, this study adopted two dimensions with a total of 6 items: learning anxiety (e.g., “AI technology updates too quickly and is very difficult to learn”) and job replacement anxiety (e.g., “I feel anxious working with AI that is smarter than me”). All items were rated on a 7-point Likert scale, ranging from 1 (strongly disagree) to 7 (strongly agree), with higher total scores indicating higher levels of AI anxiety. In this study, the Cronbach’s alpha coefficients for learning anxiety and job replacement anxiety were 0.897 and 0.908, respectively, while the coefficient of the total scale was 0.928.

#### Job crafting scale

This study adopted the Job Crafting Scale developed by Slemp and Vella-Brodrick, a commonly used tool in empirical research (Slemp and Vella-Brodrick, 2013). This scale has demonstrated good reliability among Chinese samples in prior studies (Li et al., 2023a). The scale includes task crafting (e.g., “Introduce new approaches to improve your work”), cognitive crafting (e.g., “Think about how your job gives your life purpose”), and relational crafting (e.g., “Make friends with people at work who have similar skills or interests”), with a total of 15 items. Participants rated the frequency with which they engaged in job crafting activities from 1 (hardly ever) to 5 (very often). In the present study, the Cronbach’s alpha coefficients for task crafting, cognitive crafting, and relational crafting were 0.820, 0.836, and 0.883, respectively, and the coefficient for the overall scale was 0.938.

#### Work engagement scale

The Shortened Version of the Utrecht Work Engagement Scale (UWES-9), developed by Schaufeli et al., was adopted in this study (Schaufeli et al., 2006). This scale has demonstrated good reliability in the Chinese context (Liu et al., 2025). It contains 9 items and is divided into three dimensions: vigor (e.g., “At my work, I feel bursting with energy”), dedication (e.g., “I am enthusiastic about my job”), and absorption (e.g., “I am immersed in my work”). All items were rated on a 7-point Likert scale, ranging from 1 (never) to 7 (always), with higher total scores indicating higher levels of work engagement. In the present study, the Cronbach’s alpha coefficients for vigor, dedication, and absorption were 0.927, 0.860, and 0.908, respectively, and the coefficient for the overall scale was 0.937.

#### Perceived organizational support scale

This study adopted the Perceived Organizational Support Scale (Ning, 2010), which was developed and locally validated in China by Ning. This scale consists of 10 items, divided into two dimensions: work support (e.g., “The organization provides assistance when problems arise in work”) and life support (e.g., “The organization provides assistance when employees encounter problems in daily life”). All items were scored on a 7-point Likert scale from 1 (strongly disagree) to 7 (strongly agree). Higher total scores represent higher levels of perceived organizational support. In this study, the Cronbach’s alpha coefficients for work support and life support were 0.893 and 0.883, respectively, and the coefficient for the overall scale was 0.927.

#### Demographic covariates

Gender (0 = male, 1 = female), teaching experience (1 = 3 years or less, 2 = 4–6 years, 3 = 7–9 years, 4 = 10 years or more), educational background (1 = college degree or below, 2 = bachelor’s degree, 3 = master’s degree or above), kindergarten type (0 = private, 1 = public), and professional title (1 = no professional title, 2 = primary, 3 = intermediate, 4 = senior or above) were included as covariates in this study.

### Statistical analysis

Statistical analysis of the data was performed using SPSS 27.0 and Mplus 8.3. SPSS 27.0 was used for reliability analysis, common method bias test, descriptive statistics, and correlation analysis. To verify the research hypotheses, Mplus 8.3 was employed to test the structural equation model. In the moderated mediation effect analysis, the measurement indicators of all latent variables were standardized (*Z*-scores) to reduce multicollinearity. To evaluate the model fit, commonly used fit indices were selected, including the chi-square (*χ^2^*), degrees of freedom (*df*), Comparative Fit Index (CFI), Tucker-Lewis Index (TLI), Root Mean Square Error of Approximation (RMSEA), and Standardized Root Mean Square Residual (SRMR).

## Results

### Common method bias

All data in this study were collected through teachers’ self-reports. To examine whether there was a serious common method bias, Harman’s single-factor test was adopted, and exploratory factor analysis was conducted on all items. The results showed that 6 factors with characteristic roots greater than 1 were extracted without rotation. The variance explained rate of the first factor was 32.231%, which was lower than the critical value of 40%, indicating that there was no serious common method bias in this study.

### Description statistics and correlation matrix

The means, standard deviations, and correlation coefficients of each variable are presented in Table 2. The results indicated that AI anxiety was significantly negatively correlated with work engagement (*r* = −0.320, *p* < 0.001) and job crafting (*r* = −0.434, *p* < 0.001); job crafting was significantly positively correlated with work engagement (*r* = 0.283, *p* < 0.001); perceived organizational support was significantly negatively correlated with AI anxiety (*r* = −0.192, *p* < 0.001), and significantly positively correlated with job crafting (*r* = 0.410, *p* < 0.001) and work engagement (*r* = 0.392, *p* < 0.001).

**Table 2:** Descriptive statistics and correlation matrix for each variable (*N* = 933).

Variables	1	2	3	4	5	6	7	8	9
1 Gender	1								
2 Teaching experience	0.010	1							
3 Education background	−0.048	−0.171^***^	1						
4 Type of kindergarten	−0.036	−0.086^**^	0.148^***^	1					
5 Professional title	0.005	0.754^***^	−0.191^***^	−0.149^**^	1				
6 AI anxiety	0.031	−0.015	0.037	−0.004	−0.021	1			
7 Job crafting	−0.001	−0.032	0.017	−0.054	−0.049	−0.434^***^	1		
8 Perceived organizational support	0.113^***^	0.021	−0.057	−0.007	0.022	−0.192^***^	0.410^***^	1	
9 Work engagement	0.191^***^	−0.002	−0.053	−0.021	0.005	−0.320^***^	0.283^***^	0.392^***^	1
*M*	0.916	2.199	1.829	1.429	2.168	4.367	3.293	4.434	4.783
*SD*	0.277	0.949	0.756	0.495	0.906	1.322	0.926	0.953	1.092

**p < 0.01, ***p < 0.001.

### Testing for the mediating role of job crafting

This study tested the mediating effect of job crafting in accordance with the testing procedures for structural equation mediation analysis. To examine the significance of the mediating effect, the Bias-Corrected Bootstrap method (Bootstrap = 5,000) was used to estimate the confidence interval. If the 95% confidence interval (CI) does not contain 0, it indicates statistical significance.

First, the direct effect of early childhood teachers’ AI anxiety on work engagement was tested. The model showed a good fit with *χ^2^* = 53.161, *df* = 24, RMSEA = 0.036, CFI = 0.988, TLI = 0.983, SRMR = 0.026. The results showed that after incorporating gender, teaching experience, educational background, kindergarten type, and professional title as covariates, early childhood teachers’ AI anxiety was significantly negatively associated with their work engagement (*β* = −0.367, *p* < 0.001), and the variance explained for work engagement was 17.6%. Thus, H1 was supported.

Second, job crafting was incorporated as a mediating variable for testing, and the model exhibited a good fit with *χ^2^* = 168.398, *df* = 47, RMSEA = 0.053, CFI = 0.976, TLI = 0.965, SRMR = 0.030. The results of the mediating effect test showed that the variance explained for job crafting and work engagement was 21.6 and 18.5%, respectively. AI anxiety significantly negatively predicted job crafting (*β* = −0.457, *p* < 0.001) with a 95% CI of [−0.484, −0.367], while job crafting significantly positively predicted work engagement (*β* = 0.133, *p* = 0.001) with a 95% CI of [0.047, 0.189]. The direct effect value was −0.248 (*p* < 0.001), and the mediating effect value was −0.051 (*p* < 0.001) with a 95% CI of [−0.082, −0.021], which did not include 0, indicating that the mediating effect was significant. The mediating effect accounted for 17.057% of the total effect (−0.299, *p* < 0.001). Therefore, job crafting mediated the relationship between early childhood teachers’ AI anxiety and work engagement, and H2 was supported.

### Testing of moderated mediation model

The moderated mediation effect was tested using the Latent Moderated Structural Equations (LMS) in this study. Since the LMS method does not provide traditional fit indices, the model was tested in this study following the two-step procedure (Maslowsky et al., 2015). The first step was to construct a baseline model without interaction terms. This model incorporated the main effect of perceived organizational support based on the mediating effect model where AI anxiety affects work engagement through job crafting. The results showed that the baseline model without interaction terms had a good fit: *χ^2^* = 346.017, *df* = 71, RMSEA = 0.064, CFI = 0.955, TLI = 0.940, SRMR = 0.065, AIC_0_ = 20706.556, Log Likelihood_0_ = −10309.278.

The second step was to add the latent interaction term (AI anxiety × perceived organizational support) to the baseline model to form a full model. Two methods were adopted to determine whether the model containing the interaction term had a better fit than the baseline model. The first method was based on the AIC value: if the AIC value decreased or remained unchanged, the model with the interaction term did not deteriorate. The second method employed the log-likelihood ratio test, calculating the value LR = −2[Log Likelihood_0_ − Log Likelihood_1_] and conducting a chi-square test on the results. If the result was significant, it indicated that the moderated mediation model had a better fit. The results showed that AIC_1_ = 20669.912 < AIC_0_ = 20706.556, a decrease of 36.644, indicating an improvement in the full model. Log Likelihood_1_ = −10289.956 > Log Likelihood_0_ = −10309.278, an increase of 19.322, and LR = 38.644. With the degree of freedom set as the difference in the number of free parameters between the two models (*df* = 1), the chi-square test showed *p* < 0.001, indicating a significant difference. These results indicated that compared with the baseline model, the full model containing the interaction term had a better fit, meaning the moderated mediation model was acceptable.

**Figure 2:**
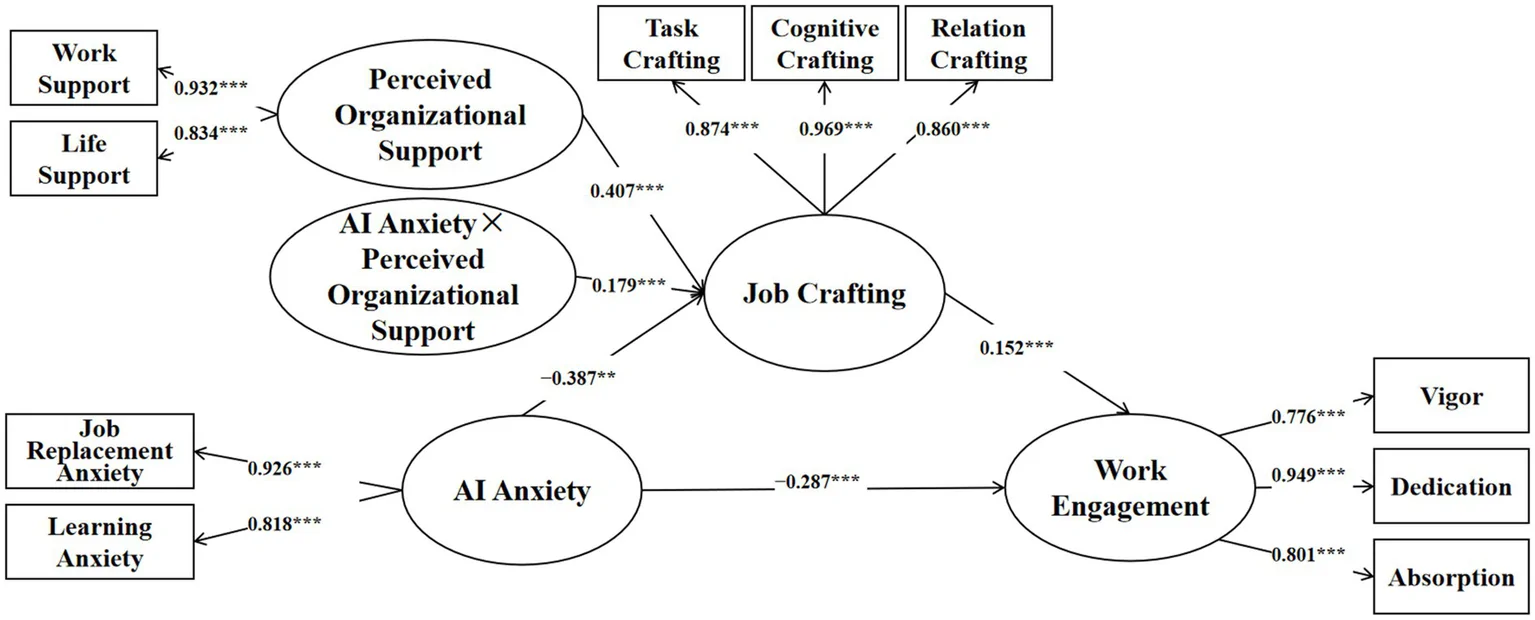
Moderated mediation analysis results. **p < 0.01, ***p < 0.001.

The results of the moderated mediation model test indicated that the interaction term of AI anxiety and perceived organizational support significantly and positively predicted job crafting (*β* = 0.179, *p* < 0.001) with a 95% CI of [0.125, 0.233]. In the high perceived organizational support group, the mediating effect value of job crafting was −0.027, with a 95% CI of [−0.043, −0.010]. In the low perceived organizational support group, the mediating effect value was −0.072, with a 95% CI of [−0.111, −0.034]. The difference in mediating effects between the two groups was 0.046, with a 95% CI of [0.018, 0.073]. None of the 95% confidence intervals contained 0, indicating that perceived organizational support significantly moderated the mediating effect of job crafting, and H3 was supported. The research results are presented in Figure 2.

**Figure 3:**
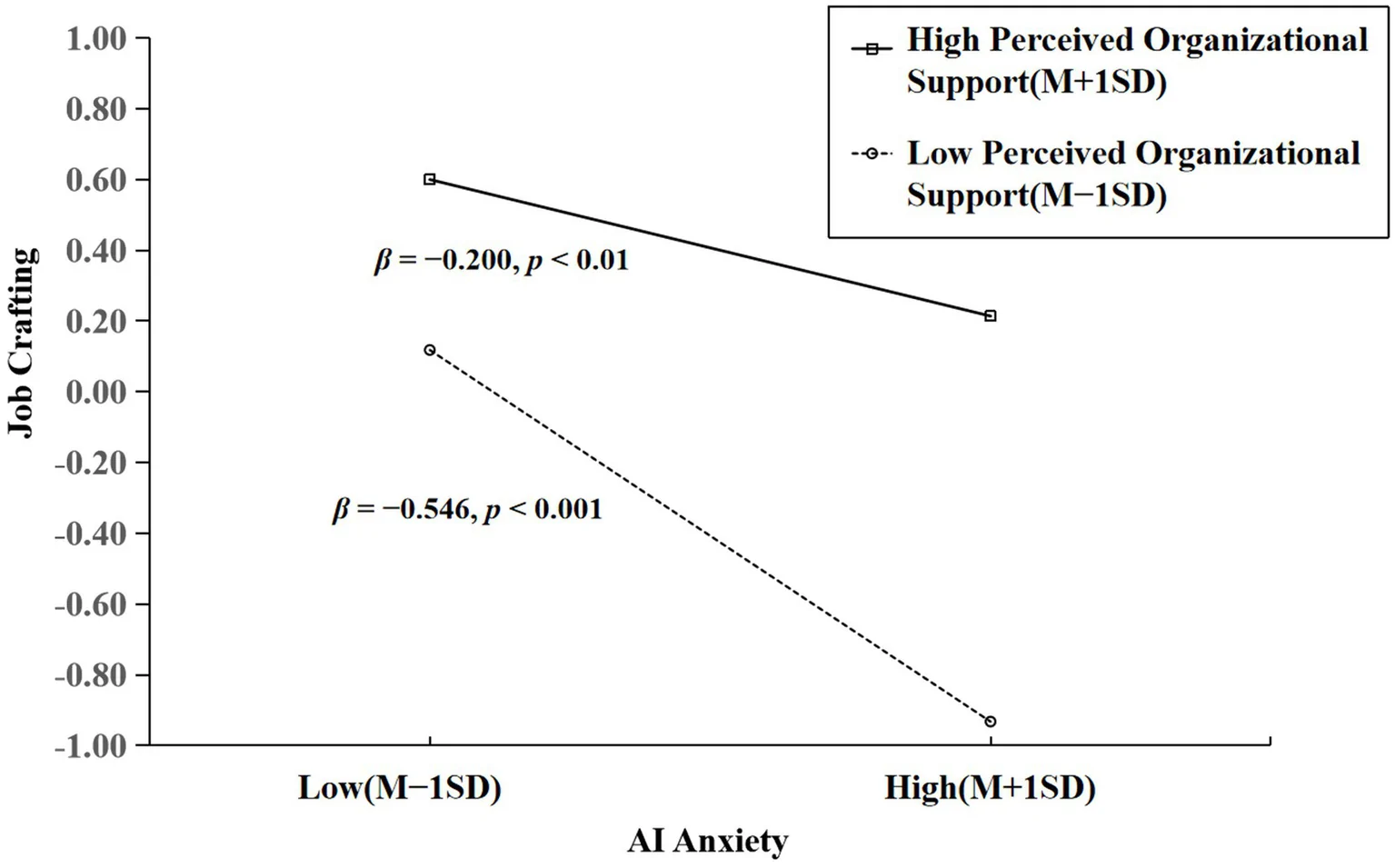
Relationship between AI anxiety and job crafting at different levels of perceived organizational support.

To further explore the moderating effect of perceived organizational support, a simple slope test was conducted. The results showed that in the high perceived organizational support group (*M + 1SD*), the negative impact of AI anxiety on job crafting was significantly weakened (*β* = −0.200, *p* < 0.01). In the low perceived organizational support group (*M − 1SD*), the negative impact of AI anxiety on job crafting was significantly strengthened (*β* = −0.546, *p* < 0.001). As can be seen from Figure 3, with the increase in AI anxiety, job crafting in the low perceived organizational support group decreased significantly, while the downward trend of job crafting in the high perceived organizational support group slowed down, and job crafting in the high perceived organizational support group was consistently higher than that in the low perceived organizational support group. This indicates that perceived organizational support played a buffering role, moderating the negative impact of early childhood teachers’ AI anxiety on job crafting.

## Discussion

### The relationship between early childhood teachers’ AI anxiety and work engagement

This study found that early childhood teachers’ AI anxiety significantly and negatively predicted their work engagement, which aligns with previous studies (Zhang et al., 2025). The application of AI in early childhood education requires teachers to adjust their teaching methods and develop new skills. Practical pressures such as the challenges posed by AI technology to teachers’ AI digital competencies (Ng et al., 2023), their limited knowledge about AI and a lack of resources (Aguilar-Cruz and Salas-Pilco, 2025), and digital teaching burnout (Duan and Zhao, 2024) lead teachers to doubt their ability to adapt to technology-integrated teaching, thereby triggering learning anxiety. In addition, the impact of AI technology on traditional teaching models may cause teachers to experience a professional identity crisis stemming from the perceived replaceability of their professional value. When their irreplaceability in the educational process is called into question, this further intensifies teachers’ job displacement anxiety. Existing studies point out that the application of AI in early childhood education is accompanied by problems such as inadequate school support, children’s weak overall competencies, teachers’ poor knowledge of AI and insufficient curriculum guidance (Su, 2024), which underscore the predicaments of early childhood teachers. Early childhood teachers constantly endure heavy childcare and educational burdens by virtue of their job characteristics, leaving them with finite psychological resources. Acting as an extra negative emotional burden, AI anxiety continuously depletes teachers’ psychological energy. When teachers excessively worry about their insufficient technical competence and fear that AI will compromise the quality of childcare and education, they will gradually lose work vigor, along with reduced professional identity and concentration. This ultimately results in lower levels of work engagement.

### Mediating role of job crafting

This study confirmed that job crafting mediated the relationship between early childhood teachers’ AI anxiety and work engagement. On the one hand, early childhood teachers’ AI anxiety significantly and negatively predicted job crafting. The transformation and impacts of AI technology on education have led early childhood teachers to perceive threats to their professional competencies, occupational value and job security. Such perceptions further translate into job insecurity, which impedes the practice of job crafting (Xu F. et al., 2026). Meanwhile, early childhood teachers’ recognition of the opportunities brought by AI development tends to be offset by their internal sense of threat, creating additional barriers to the advancement of job crafting. When teachers suffer from AI anxiety, their cognition remains fixated on the threats posed by AI technology. Their limited psychological resources are primarily devoted to coping with perceived threats and regulating negative emotions. Consequently, there is a severe shortage of psychological resources available for actively adjusting work patterns, resulting in a decline in job crafting behaviors. On the other hand, job crafting significantly and positively predicted early childhood teachers’ work engagement, which was consistent with the findings of previous studies (Huang X. et al., 2025; Shang, 2022). According to the COR theory (Halbesleben et al., 2014), individuals will adopt defensive strategies and rationally allocate remaining resources to prevent continuous resource loss once they perceive that their resources are at risk of depletion. When early childhood teachers view educational technological changes from a challenge perspective and aim to improve their own competencies, they will proactively explore growth opportunities in the environment and engage in job crafting. By adjusting job tasks, optimizing interpersonal interactions and reshaping cognitive patterns, early childhood teachers gradually strengthen their sense of work control and autonomy, thereby boosting their work engagement. Conversely, if teachers lack effective job crafting behaviors, the negative emotions triggered by AI anxiety will keep accumulating and continuously deplete their work enthusiasm, which is detrimental to their work engagement.

### Moderating role of perceived organizational support

This study found that perceived organizational support significantly moderated the relationship between AI anxiety and job crafting. Specifically, high perceived organizational support weakened the inhibitory effect of AI anxiety on job crafting; in contrast, low perceived organizational support exacerbated the negative impact of AI anxiety on job crafting, thereby affecting work engagement. Perceived organizational support is an important external resource perceived by individuals and can mitigate the negative effects of work-related stress (Liang et al., 2022). Organizational support provided teachers with resource guarantees to cope with AI anxiety, such as AI skill training, psychological counseling, and work adjustment guidance. This reduced teachers’ concerns about their insufficient abilities and thus enhanced their confidence and capacity to proactively engage in job crafting. When teachers felt recognized by the organization and their efforts valued, they were more inclined to adopt creative teaching approaches (Huang M. et al., 2025). Even when experiencing a certain level of anxiety, teachers are more inclined to cope with changes through job crafting, which contributes to their sustainable career development (Li et al., 2025). Conversely, low perceived organizational support intensified the dual pressures of threat perception and resource scarcity, amplifying the negative effect of AI anxiety on job crafting. Without systematic, professional guidance, training, and organizational support, teachers could neither effectively acquire relevant resources for technology application to alleviate competence anxiety nor easily construct positive perceptions of AI technology empowering teaching. Instead, this reinforced negative cognitions and further aggravated AI anxiety. In environments with inadequate organizational support, teachers have to bear the pressure brought by AI technology on their own. They can hardly conduct effective job crafting, and the negative effects of AI anxiety on work engagement will be further intensified.

## Implications

To enhance early childhood teachers’ work engagement, proactive attention should first be paid to their emotional states to alleviate their AI anxiety. It is necessary to conduct regular communication and in-depth listening to learn about the practical difficulties teachers face during the intelligent transformation of preschool education. Meanwhile, targeted cognitive guidance and professional training should be delivered to help teachers view AI rationally. It should be clarified that AI acts as an auxiliary tool to support childcare and education, rather than a potential threat to teachers’ professional development, so as to eliminate their resistance and panic. Furthermore, teachers’ psychological capital should be steadily consolidated to build their confidence and skills for addressing challenges, thereby inspiring work enthusiasm and raising the overall level of work engagement.

Second, conscious guidance and motivation should be provided to encourage early childhood teachers to engage in job crafting. This involves fostering teachers’ intrinsic motivation for job crafting (Polatcan et al., 2026) and encouraging them to adjust their workload according to personal needs, traits and energy levels. Supported by teaching research seminars and exchanges of outstanding cases, teachers are encouraged to revise daily childcare and education routines based on children’s individual traits, and explore effective ways to integrate AI into play-based instruction. Additionally, when teachers are granted pedagogical autonomy to adjust the application of AI tools according to instructional demands, their AI anxiety can be converted into enthusiasm for exploration and creation. This helps teachers shift from passive recipients of technology to active integrators and innovators, enabling them to optimize work processes and reconstruct professional value through job crafting.

Finally, it is necessary to perfect the organizational support system and strengthen early childhood teachers’ perceived organizational support. Tiered and targeted AI training programs can be developed, which help avoid burdening teachers with overly complex technical content. Organizations ought to provide more tolerance and support, build trust with teachers, grant rational authorization, and maximize the role of individual job crafting (Hu et al., 2024). Sufficient resources should be offered to teachers to cope with technological transformation, thereby buffering the negative impact of AI anxiety and enhancing their work engagement. Meanwhile, efforts should be rooted in sustainable education goals to promote the integration of AI and humanistic care in preschool education. The core people-oriented principle of early childhood education should be upheld, and the auxiliary role of AI should be clarified. AI should be utilized to support children’s development and reduce teachers’ workloads, thereby realizing the coordinated and sustainable development of AI technology, teachers and children.

## Limitations and future research

This study has the following limitations. First, it adopted a cross-sectional design, which cannot determine the causal relationships between variables. Future research may use a longitudinal design to examine the long-term dynamic relationships among AI anxiety, job crafting, and work engagement. Second, in terms of sample selection, the sample representativeness is relatively limited and may not fully represent the situation of all early childhood teachers. Future research can further expand the sample size to cover early childhood teachers from more regions and types of kindergartens, so as to improve the universality and representativeness of the research results. Third, this study focused on the mediating role of job crafting and the moderating role of perceived organizational support. Future research can further explore other mediating variables (e.g., professional identity and self-efficacy) and moderating variables (e.g., teachers’ age and technical literacy) to construct a more comprehensive mechanism model. Finally, data collection mainly relied on self-reports. Future research can combine observation methods, interview methods, and objective performance data to enhance the objectivity of the research results.

## Conclusion

This study demonstrated the negative relationship between AI anxiety and work engagement among early childhood teachers, and revealed the mediating role of job crafting and the moderating role of perceived organizational support. The findings indicated that to improve early childhood teachers’ work engagement, it is necessary to alleviate their AI anxiety, strengthen their perceived organizational support, and cultivate job crafting. This study proposed practical implications for alleviating early childhood teachers’ AI anxiety and improving their work engagement, and carries important practical implications for facilitating their sustainable professional development.

## Data Availability

The original contributions presented in the study are included in the article/supplementary material, further inquiries can be directed to the corresponding author.
